# Miniaturized Swimming Soft Robot with Complex Movement Actuated and Controlled by Remote Light Signals

**DOI:** 10.1038/srep17414

**Published:** 2015-12-03

**Authors:** Chaolei Huang, Jiu-an Lv, Xiaojun Tian, Yuechao Wang, Yanlei Yu, Jie Liu

**Affiliations:** 1 State Key Laboratory of Robotics, Shenyang Institute of Automation, Chinese Academy of Sciences, Shenyang 110016, China; 2 Department of Materials Science & State Key Laboratory of Molecular Engineering of Polymers, Fudan University, Shanghai 200433, China; 3 Department of Chemistry, Duke University, Durham, North Carolina 27708, USA; 4 University of Chinese Academy of Sciences, Beijing 100049, China

## Abstract

Powering and communication with micro robots to enable complex functions is a long-standing challenge as the size of robots continues to shrink. Physical connection of wires or components needed for wireless communication are complex and limited by the size of electronic and energy storage devices, making miniaturization of robots difficult. To explore an alternative solution, we designed and fabricated a micro soft swimming robot with both powering and controlling functions provided by remote light, which does not carry any electronic devices and batteries. In this approach, a polymer film containing azobenzene chromophore which is sensitive to ultra-violet (UV) light works as “motor”, and the UV light and visible light work as “power and signal lines”. Periodically flashing UV light and white light drives the robot flagellum periodically to swing to eventually push forward the robot in the glass tube filled with liquid. The gripper on robot head can be opened or closed by lights to grab and carry the load. This kind of remotely light-driven approach realizes complex driving and controlling of micro robotic structures, making it possible to design and fabricate even smaller robots. It will have great potential among applications in the micro machine and robot fields.

To drive a machine or a robot to perform specific function, we must provide it with power and control information. Generally speaking, from common machines used in our daily life to the widely used industry robots, most of them are driven by electrical motors. The control information transports through signal lines or wireless communication, and the energy transports through power lines or comes from electric battery loaded on the machine or the robot. However, for the robot at millimeter size or smaller, it becomes difficult to load a battery with enough capacitance and the wires could limit its movement if connected from external power source^1^. How to provide energy and transport control information to the robots become two key problems in designing and fabricating a micro machines or robots.

To explore solutions to these problems, one possible approach is to obtain energy from its surrounding environment, such as using cardiomyocytes or cardiac cells to drive the micro machine by getting energy from nutrient solution^2^,^3^. Another approach is to remotely supply energy and transport control information to the robot, examples include temperature field^4^,^5^, magnetic field^6^,^7^,^8^,^9^,^10^,^11^, electric field^12^,^13^ and etc. On the other hand, light is another energy source that has not been widely studied to drive and control the motion of robots. There is very little progress due to the lack of suitable transform mode from light energy to mechanical energy, except for optical tweezers which used lasers energy to trap micro particle^14^, or photons to collide and move micro particle^15^.

With the development of material science in recent years, some materials with special functions are discovered and synthesized, which can convert other kinds of energy, such as electrical energy^12^,^13^,^16^, magnetic energy^17^,^18^ and heat^19^,^20^, into mechanical energy and thus avoid the complex drive and transmission mechanisms. Using those materials, several new kinds of micro robots were fabricated, such as earthworm-like micro-robot^21^ and fish-like micro-robot^22^ made from shape memory alloy, walking micro-robot with cilia-like thermal bimorph actuator^4^, fly-like flapping-wing micro robot driven by piezoelectric actuators^1^, gel walkers made from electro-actuated hydrogel^13^, micro inchworm robot actuated by electro-conjugate fluid^16^ etc. As they are directly driven by electrical energy or convert electrical energy to other types of energy for driving, they all need power lines or batteries to supply energy. On the other hand, ferroelectric ceramics (Pb, La)(Zr, Ti)O_3_ (PLZT) was discovered with photostrictive effect^23^ and several PLZT actuators driven by lights were fabricated^24^,^25^. While, influenced by the poor photostrictive effect, PLZT actuators have had very limited applications in nearly recent twenty years since 1997^26^.

With the invention of light-driven liquid-crystal material^27^, some preliminary light-driven devices have been fabricated as the material can directly convert light energy into mechanical energy with fast response, large deformation and more than hundred thousand cycles’ deformation and recovery^28^, such as simple elastomer^29^, deformable 3D microstructure^30^, and even spring^31^ etc. In our approach, we use this light-driven material to fabricate a miniaturized swimming soft robot with complex movement and function. At the same time, control information is embedded into the light beam to realize robot driving and controlling. This approach greatly simplified the design of the moving robot, making it possible to use light-sensitive materials in micro robots capable of realizing complex functions. Here, as a demonstration of this principle, light signals are used to drive and control a micro swimming robot, which could also grab and release the load with a light-driven gripper.

## Light-driving material

**Figure 1:**
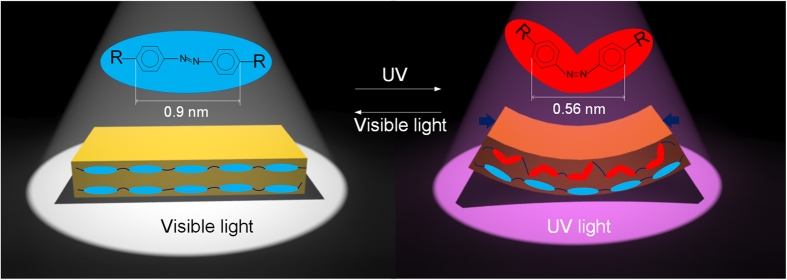
Transformation of trans/cis isomeric azobenzene and LDLCF. The length of trans-isomeric azobenzene is 0.9 nm and it will convert to cis-isomeric azobenzene with length 0.56 nm when irradiated by UV light, it will recover to its original state when irradiated by white light. As the top surface of LDLCF will mostly absorb UV light due to its strong absorption, LDLCF will bend other than shrink.

Azobenzene chromophore has cis-trans isomerism and the two isomers can be converted to each other when they are irradiated by ultraviolet (UV) light and visible light. As the two isomers have different length, UV and visible light irradiation can change the length of azobenzene chromophore. Here, light-driven liquid-crystal film^27^,^32^ (namely LDLCF) containing azobenzene chromophores is used (its detailed preparation information is shown in **Materials and Method)**, which can be bent by UV and recovered by visible light as shown in Fig. 1.

As the phase transition of azobenzene chromophore is very quick in less than 200 μs^33^, the deformation of the film is almost synchronous with light irradiation. Therefore, the film deformation can be controlled in real-time by the lights.

## Swimming robot with gripper

**Figure 2:**
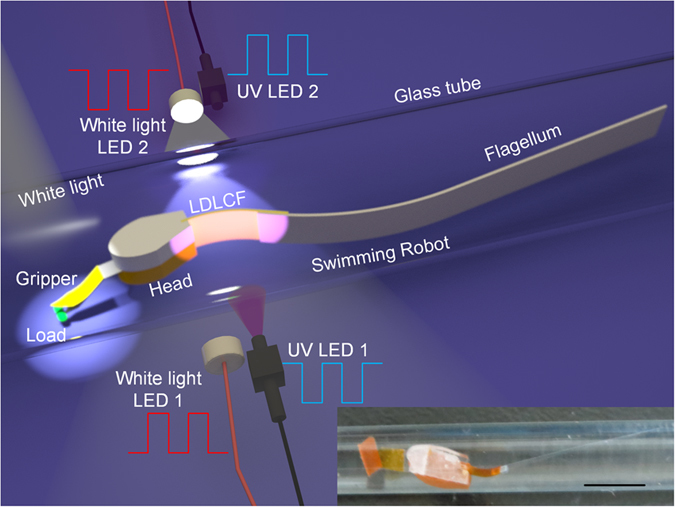
Schematic diagrams of the micro swimming robot and light irradiation system. The bottom insert at the right side of the figure is the actual robot with the load grabbed by gripper (Details of the robot fabrication process is shown in Materials and Methods) and the scale bar is 5 mm. The light beam of controlling the gripper is perpendicular to that of driving the robot to swim. The head of micro swimming robot has width 2 mm, height 3 mm and length 5.5 mm, and the whole robot has length 26.3 mm and weight 31.9 mg.

In nature, microorganism swims in liquid mainly by two modes with either a rotating helix-shaped or a swinging flexible oar-like flagellum^34^. Inspired by this, we designed a micro swimming robot with a head and a flexible long flagellum in liquid-filled tube with a low Reynolds number (*Re*) as shown in Fig. 2. Although its whole length is 2.6 cm, its swimming mechanism is similar to microorganism with a swing flexible oar-like flagellum, such as choanoflagellate or spermatozoon. Additionally, a gripper is added on the robot head to introduce a function to grab and release loads. The robot swimming is driven and controlled by 4 remote light sources, which are 2 UV LEDs and 2 white-light LEDs at both sides as shown in Fig. 2.

The robot flagellum is made up of flexible polymer and performs wave-like swing under the LDLCF driving force and viscous resistance. As the viscous coefficients parallel and perpendicular to the flagellum are different, the resultant viscous force of the flagellum has a component force, namely the propulsive force at the forward direction, which drives the robot to swim forward^34^,^35^. Average propulsive force is about 1.11 μN and it balances the viscous resistance and frictional resistance of the head when the robot swims with speed 142 μm/s (Details of force analysis and calculation are shown in Supplementary Information).

The gripper is fixed on the head of the robot, and it is made of movable LDLCF and stationary polyethylene terephthalate (PET). The LDLCF can be bent to open the gripper by UV light and recovered to close the gripper by white light, thus to realize the grab function as shown in Fig. 2 inserts, and the grabbing force is dependent on the load size and young’s modulus of the LDLCF and PET (Details of the clamping force calculation are shown in Supplementary Information).

As the gripper and swim-driving part are all driven by lights, their suitable space arrangement is important to avoid light interference. Here the LDLCF surface of the gripper is arranged perpendicularly to that of the swim-driving part, thus the driving light beams are also perpendicular to each other to successfully avoid the light interference as shown in Fig. 2.

## Results and Discussion

**Figure 3:**
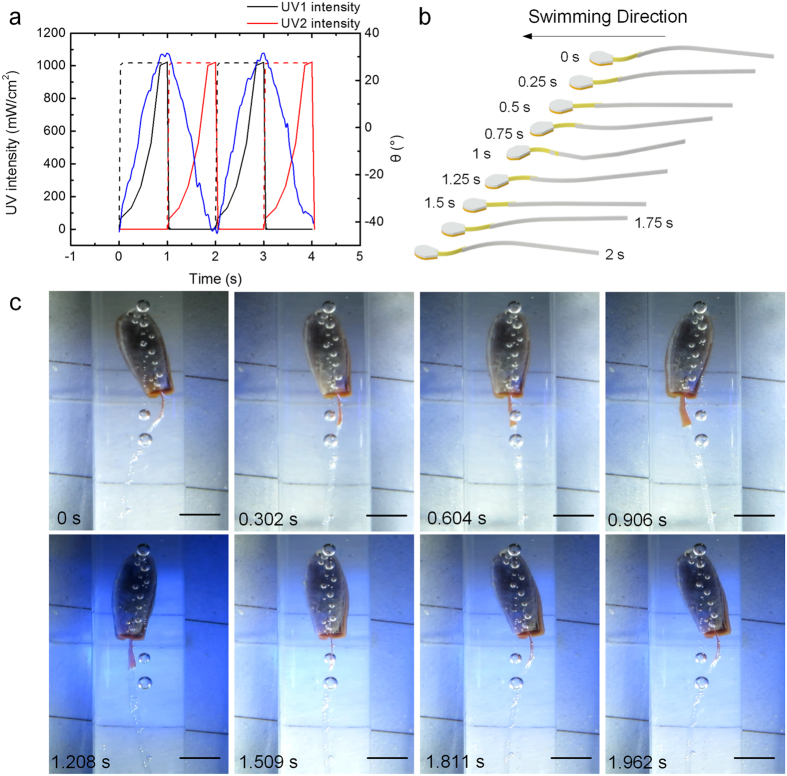
Lights drive the flagellum swing. (**a**) Changes of the UV light intensity, controlling signals and LDLCF bending angle θ in two periods. The black and red lines are light intensity of UV 1 and UV 2 respectively, and the dash lines and blue line are controlling signals and LDLCF bending angle θ. The light intensity is measured at 3 cm away from LED head. (**b**) Schematic diagram of swimming. (**c**) Periodic swing of the flagellum in a cycle. The LDLCF periodically bends and drives the flagellum periodically swing. The scale bar is 2 mm.

The UV light irradiated on the LDLCF generates contraction stress to bend the LDLCF and the white light irradiated on the LDLCF releases the contraction stress to recover the LDLCF, so a UV LED always works synchronously with opposite white light LED to accelerate the movement of the LDLCF. In the first half cycle, UV LED 1 and white light LED 2 works at the same time, LDLCF bends to UV LED 1 side. In the second half cycle, UV LED 2 and white light LED 1 works at the same time, and LDLCF bends to UV LED 2 side. These two half cycles form a whole driving cycle to actuate the robot flagellum to swing periodically as shown in Fig. 3.

It can be seen in Fig. 3(a) that as the UV LEDs intensity increases, the LDLCF bends towards the direction of the incoming light and the maximum bend angle is −44° and 32°. Periodically flash the LEDs bends the LDLCF periodically, which actuates the flagellum to swing periodically as shown in Fig. 3(b,c) (More details can be seen in Supporting Video 1). Consequently, the robot moves forward as the results of the flagellum swinging.

**Figure 4:**
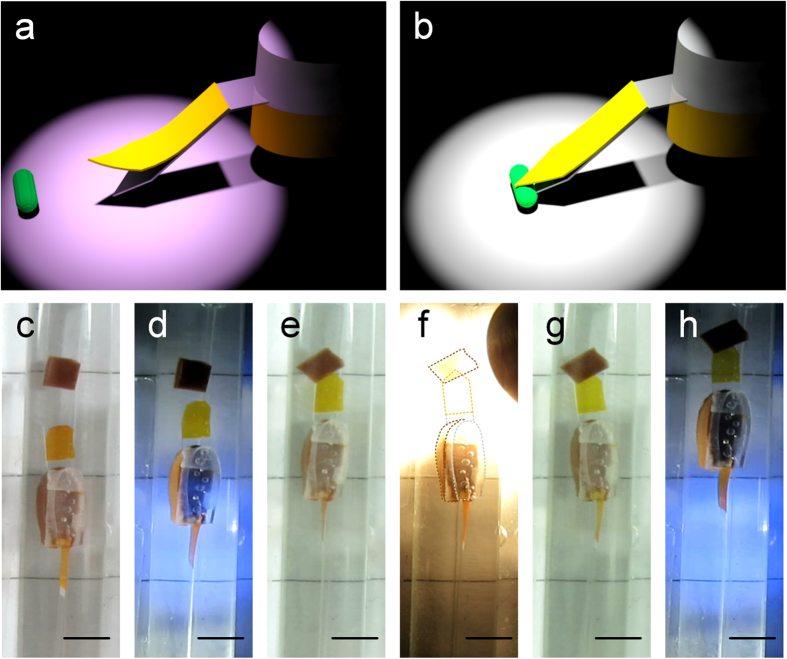
The robot with gripper grabs, carries and transports the load. (**a,b**) are the schematic diagrams of gripper opened by UV light and closed by white light. (**c**) The gripper is opened by UV light irradiation. (**d**) The flashing LEDs drive the robot to swim to the load. (**e**) The robot arrives at the load position. (**f**) White light irradiates the gripper to close it. (**g**) The load is grabbed by the gripper. (**h**) The robot carries and transports the load to the target position. The scale bar is 3 mm. In Fig. 4(f) the front half of the robot is highlighted by dotted lines to help viewing its actual position.

With the gripper assembled on the swimming robot head, the load grab and transport functions can be realized as shown in Fig. 4. Firstly the UV light irradiates the gripper to open it, and then the robot is driven to swim to the load position, where the white light irradiates the gripper to close it and grab the load. After that, the robot carries and transports the load to its target position (More details can be seen in Supporting Video 2).

**Figure 5:**
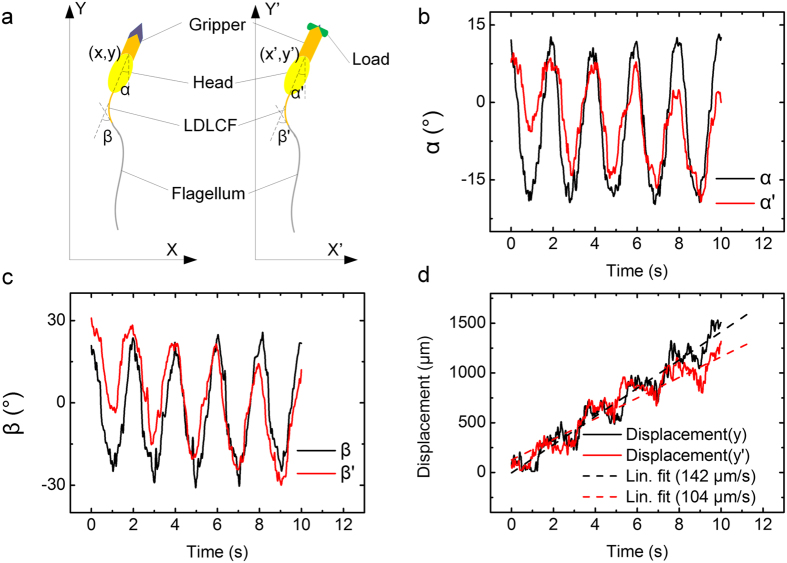
Movement analysis of the robot with gripper before and after grabbing load (**a**) Schematic diagram of the robot with gripper before and after grabbing load. Swimming direction is parallel to Y-axis. The angles between the head and the Y/Y′-axis are α and α′ and bend angles of LDLCF are β and β′. The angle marked apostrophe means the data is measured after grabbing load. The positions of the head before and after grabbing load is denoted as (x, y) and (x′, y′) in their respective coordinate system. (**b**) Angle α and α′ versus time. Amplitude of α is stable but amplitude of α′ drastically changes. At the first the amplitude of α′ is −5.9° ~ 10° and at the end the amplitude of α′ is −18° ~ 2.3°. The head direction has significant change. (**c**) Angle β and β′ versus time. Amplitude of β is −26.4° ~ 20.8° and it keeps stable. Amplitude of β′ changes significantly from −2.9° ~ 30.8° to −30.3° ~ 12° which shows obvious unbalance. (**d**) Displacement of the robot along Y axis before and after grabbing load versus time. The values of average swimming speed are respectively 142 μm/s and 104 μm/s. The dash lines in (**b,c**) are amplitude of the angles, and the dash lines in (**d**) are fitting lines.

To analyze the load carry and transport movement, data is acquired from each frame of the Supporting Video 2, and the bending angle of LDLCF, angles between the head and Y-axis, and the trajectories of the swimmer head are all plotted in Fig. 5.

The detailed movement of the robots can be obtained from careful analysis. For this specific robot shown in Fig. 5, before grabbing the load, the maximum bending angle is relatively stable, and the robot moves at a nearly constant speed of 142 μm/s as shown in Fig. 5(d). Additionally, after grabbing the load, it can be seen that the maximum amplitude of LDLCF bending angle β′ decreases slightly compared with β shown in Fig. 5(c), and the swim speed reduces slightly from 142 μm/s to 104 μm/s. A possible explanation is that when the robot swims away from the center of light irradiation, the light intensity decreased since light used here is converging light and its effective area is limited, which decreases the maximum bend angle and contributes to the lower speed. At the same time, it can also be seen from Fig. 5(b) that the robot swims closer to one side of the glass tube with the LDLCF bend angle β′ leaning to the same side as shown in Fig. 5(c), which means that the balance of light irradiation intensity from the two sides is broken after it swims away from the center of irradiation. From the experimental results, it can be seen that the swimming speed is dependent on the maximum amplitude of LDLCF bending angle which can be adjusted and controlled by the light intensity. Meanwhile, we can also purposely control and turn the swimming direction by adjusting the light intensity of one side to break the balance. In the future, we will use natural sunlight to drive the robot as UV can be got from sunlight by a light splitting system, or use laser instead of LED, to enlarge the light intensity to eventually improve the robot and also to drive the robot at a far distance as laser can keep its property over a long distance.

In summary, we fabricate this light-driven micro swimming robot with gripper based on new functional material, to realize the complex movements like swimming, grabbing, carrying and transportation. As the robot has “hand” to capture and transport objects and it is driven and controlled by lights without any lines and electromagnetic noise, it can be used in long and narrow liquid environment such as in microfluidic device and system, or in electromagnetic noise sensitive situation, to play a versatile role for capture, transportation, manipulation etc. The experimental results prove that this light-driven mode can realize non-contact energy supply, driving and complex movement control. As this approach doesn’t need any battery or power line, information processing unit and lines or any motor and transmission mechanisms, it greatly simplify the structure of the robots. Such simple and micro robots will have great potential among applications in the micro machine and robot fields.

## Materials and Method

### Design of the swimming robot

There exists two common swimming modes in microscopic world^35^. Choanoflagellate or spermatozoon has a long and flexible flagellum and swings the flagellum to generate propulsive force for swimming, while many bacteria rotate helix-shaped flagellum to generate propulsion for swimming, as shown in Supporting Figure 1(a,b).

As light-driven liquid-crystal film (namely LDLCF) can be bent and recovered by UV light and visible light, we decided to use LDLCF to drive swimming robot flagellum swinging like Choanoflagellate or spermatozoon in the microscopic word and also use LDLCF to make a gripper on the robot head, thus we designed the swimming robot as shown in Supporting Figure 1(c).

### Preparation of the light-driven liquid-crystal film (LDLCF)

DA11AB (azo-liquid-crystal monomer) and C9A (cross-linker) were synthesized and purified according to the literature^36^,^37^ and their chemical structures are shown in Supporting Figure 2. Irgacure 784 (initiator) was purchased from commercial suppliers and used as received. Differential scanning calorimetry (DSC) characterizations of DA11AB and the mixture of DA11AB and C9A are shown in Supporting Figure 3.

The mixed precusor of DA11AB and C9A in the molar ratio of 1:4 was solved in chloroform to form a solution. Then, adequate initiator was added and fully stirred in the precursor solution in a dark room. Well-distributed precusor powder (colored in orange) was obtained by slow and exhaustive evaporation of the solvent at 23 °C overnight. The melt of the precusor powder was injected into a cell with a 20 mm thick gap in isotropic phase. The inner surfaces of the cell had been coated with a rubbed polyimide layer to obtain a homogeneous alignment of the mesogens. The temperature of the cell was gradually decreased from 105 °C to 93 °C at an annealing speed of 0.1 °C·min^−1^, which was regulated by a high-precision central controller (METTLER-TOLEDO FP90). The annealed monomer mixture was in a nematic phase and cross-linked by photopolymerization at >540 nm with a high pressure Hg lamp (Beijing CHANGTUO CHF-XM250) through glass filters for 2.5–3 h (2.1–2.5 mW·cm^−2^). After the photopolymerization, the cell was opened, and the film was removed from the cell with a cutter. Polarizing optical microscope (POM) observations of the film are shown in Supporting Figure 4.

### Method to fabricate the swimming robot with gripper

The swimming robot is made up of gripper, head, LDLCF and flexible flagellum. The head contains two layers. The upper layer is pure polydimethylsiloxane (PDMS), and the bottom layer is PDMS with Fe_3_O_4_ used to increase its density. Therefore, gravity center of the swimming robot is shifted to the bottom layer to keep the swimming robot posture more stable. The processes to fabricate the head of the swimming robot are shown in Supporting Figure 5.

Mold is engraved by engraving machine, and then PDMS pre-polymer is injected in the mold which is coated with sodium dodecyl sulfate (SDS). Put the mold with PDMS pre-polymer in furnace at 80 °C for 6 h to be solidified and then get upper layer of the head. The same process is used to produce the bottom layer of the head.

The flagellum is made of polyethylene (PE) or polyethylene terephthalate (PET) film. The gripper is made up of general polymer PET and LDLCF, and it is fixed on the head of the swimming robot. All the parts of the swimming robot were assembled by adhesive materials, as shown in Supporting Figure 6.

## Additional Information

**How to cite this article**: Huang, C. *et al.* Miniaturized Swimming Soft Robot with Complex Movement Actuated and Controlled by Remote Light Signals. *Sci. Rep.* **5**, 17414; doi: 10.1038/srep17414 (2015).

## Supplementary Material

Supporting Video 1

Supporting Video 2

Supplementary Information
