# Supplementary material for: Miniaturized Swimming Soft Robot with Complex Movement Actuated and Controlled by Remote Light Signals
Source: Sci Rep. 2015 Dec 3;5:17414. doi: 10.1038/srep17414 (PMC4668837; doi:10.1038/srep17414)
Supplement: Supplementary Information [file srep17414-s3.pdf]

Supplementary Information

Miniaturized Swimming Soft Robot with Complex Movement Actuated and Controlled by Remote Light Signals

Chaolei Huang[1,4]‡, Jiu-an Lv[2]‡, Xiaojun Tian[1]*, Yuechao Wang[1], Yanlei Yu[2]*, Jie Liu[3]*

1 State Key Laboratory of Robotics, Shenyang Institute of Automation, Chinese Academy of Sciences, Shenyang 110016, China.

2 Department of Materials Science & State Key Laboratory of Molecular Engineering of Polymers, Fudan University, Shanghai 200433, China.

3 Department of Chemistry, Duke University, Durham, North Carolina 27708, USA.

4 University of Chinese Academy of Sciences, Beijing 100049, China.

*E-mail: xjtian@sia.cn, ylyu@fudan.edu.cn, j.liu@duke.edu

‡ These authors contributed equally to this work.

Forces analysis and calculation during swimming

The swimming robot was put in a glass tube filled with liquid which has 5 mm internal diameter. During swimming, the interaction between flexible flagellum and liquid generates propulsive force to push forward the swimming robot. Na_2SO_4 is added in the water to adjust density of the liquid. As the density of the liquid is 1.022×10^3 kg/m^3, and the swimming robot's weight and volume are 31.9 mg and 30 mm^3 respectively, the swimming robot's density is 1.0633×10^3 kg/m^3 which is a little large r than the liquid density.

As average speed v of the swimming robot in experiment is 142 μm/s, the head section area S is 6 mm^2, parameter of the head section P is 10 mm and dynamic viscosity of the fluid μ is about 10^{-3} Pa s, so the Reynolds number of the swimming robot in the glass tube is $Re = \frac{\rho v D_H}{\mu} = \frac{\rho v 4S/P}{\mu} = 0.348$ [1]. With the low Reynolds number, the swimming robot has a similar movement mechanism with spermatozoon or choanoflagellate in fluid.

As shown in Supporting Figure 7, ds is a part of flagellum's length s and it has angle $\theta(s,t)$ to Y axis and velocity $v(s,t)$. The viscous coefficients parallel and perpendicular to the ds are $\zeta_\perp$ and $\zeta_{/\!/}$, so the viscous drag force f of ds is

$$f = -\left(\zeta_\perp v(s,t)_\perp + \zeta_\parallel v(s,t)_\parallel\right)\mathrm{d}s, \tag{1}$$

Where $v(s,t)_\perp$ and $v(s,t)_\parallel$ are the projections of the velocity $v(s,t)$ on the directions parallel and perpendicular to the ds. With relationship $v(s,t)_\perp = v(s,t)\sin[\theta(s,t)]$ and $v(s,t)_\parallel = v(s,t)\cos[\theta(s,t)]$, we can get the forward propulsive force F_p [2]

$$F_\mathrm{p} = (\zeta_\perp - \zeta_\parallel)v(s,t)\sin[\theta(s,t)]\cos[\theta(s,t)]. \tag{2}$$

For a flagellum has length l, the average propulsive force in a period T is

$$\overline{F_p} = \frac{\int_0^\mathrm{T}\int_0^\mathrm{l}(\zeta_\perp - \zeta_\parallel)v(s,t)\sin(\theta(s,t))\cos(\theta(s,t))\mathrm{d}s\mathrm{d}t}{\mathrm{T}}. \tag{3}$$

From equation (3), in order to generate net propulsion from a time-periodic movement, both the velocity $v(s,t)$ and angle $\theta(s,t)$ need to vary periodically in time.

When the swimming robot moves in the liquid, it will be affected by gravity G, buoyancy F_b,

force N and friction force f_r between the swimming robot and glass tube wall, viscous force f_v in liquid and propulsive force F_p. The forces analysis is shown in Supporting Figure 8(b). When the swimming robot has a uniform velocity $\bar{v}$, we can get the relationship,

$$\bar{F_p} = f_v + f_r, \tag{4}$$

$$G = N + F_b, \tag{5}$$

where $f_r = \mu N$, $f_v = C_1 \bar{v}$, μ and C_1 are coefficient of friction and drag constant.

The swimming robot's weight and volume are m and V respectively, and liquid density is ρ, so we can get average propulsive force,

$$\bar{F_p} = \mu g(m - \rho V) + C_1 \bar{v}. \tag{6}$$

As the friction coefficient μ is 0.014 and drag constant C_1 is 6.61×10^{-3} N s/m from experimental measurement, the average propulsive force will be 1.11 μN when the average velocity $\bar{v}$ is 142 μm/s.

Forces analysis and calculation during clamping

The two layer gripper consists of LDLCF and general polymer PET. During grabbing, the gripper is closed when it is irradiated by visible light. After irradiated by visible light, there will be no UV-induced stress left in the LDLCF, thus the grabbing force comes from the deformation of LDLCF and PET. To calculate the grabbing force, we can use cantilever beam model as shown in Supporting Figure 9.

The movable end LDLCF and fixed end PET have same length l and width b. Their thickness are t_1 and t_2, elastic modulus are E_1 and E_2, and so their bending stiffness are $E_1bt_1^3/12$ and $E_2bt_2^3/12$ respectively. The bending deflections y_1 and y_2 can be expressed as,

$$y_1 = \frac{Fl^3}{3EI} = \frac{4Fl^3}{E_1bt_1^3}, \tag{7}$$

$$y_2 = \frac{Fl^3}{3EI} = \frac{4Fl^3}{E_2bt_2^3}, \tag{8}$$

where F is the clamping force and I is the area moment of inertia. As we known y_1+y_2=D, the clamping force is,

$$F = \frac{E_1E_2bDt_1^3t_2^3}{4l^3(E_1t_1^3+E_2t_2^3)}. \tag{9}$$

From equation (9), we can see the grabbing force linearly increases with D of the grabbed object and young's modulus of the LDLCF and PET.

As parameters of the gripper are E_1=90 MPa, E_2=2.25 GPa, b=1.5 mm, t_1=35 μm, t_2=30 μm, l=5 mm, the grabbing force will be 10.89 μN when the grabbed object's diameter or thickness is 1 mm.

Supporting references

1 Wikipedia, Reynolds number, https://en.wikipedia.org/?title=Reynolds_number, accessed: June, 2015.

2 Yu, T. S., Lauga, E. & Hosoi, A. E. Experimental investigations of elastic tail propulsion at low Reynolds number. *Phys Fluids* **18**, 091701 (2006).

Supporting Video 1 A video to show the flagellum swings periodically.

Supporting Video 2 A video to show the swimming soft robot swims forward and carries a load.

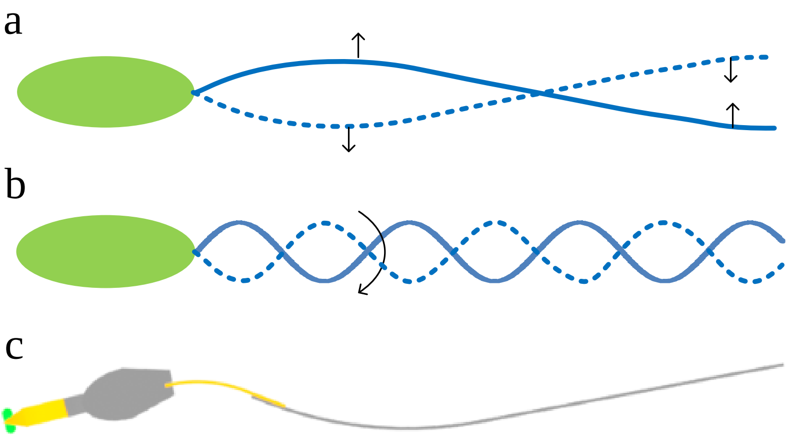

Supporting Figure 1 Design of the swimming robot and two swimming modes in microscopic world. (a) Swing slender flagellum to swim in fluid. (b) Rotate helix-shaped flagellum to swim in fluid. (c) Top view of the designed swimming robot (yellow parts are LDLCF).

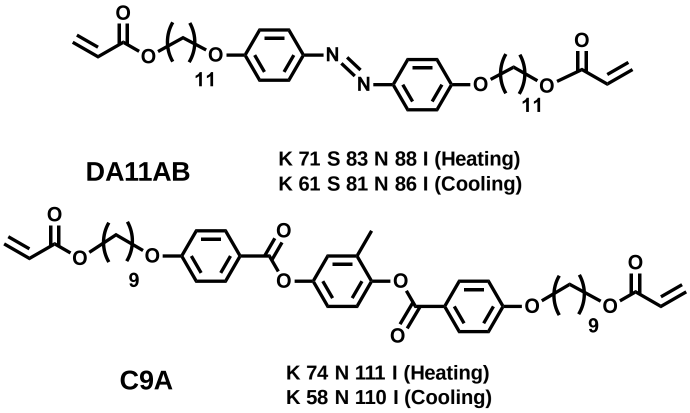

Supporting Figure 2 Chemical structures and properties of DA11AB and C9A used in this study. K, crystal; S, smectic; N, nematic; I, isotropic.

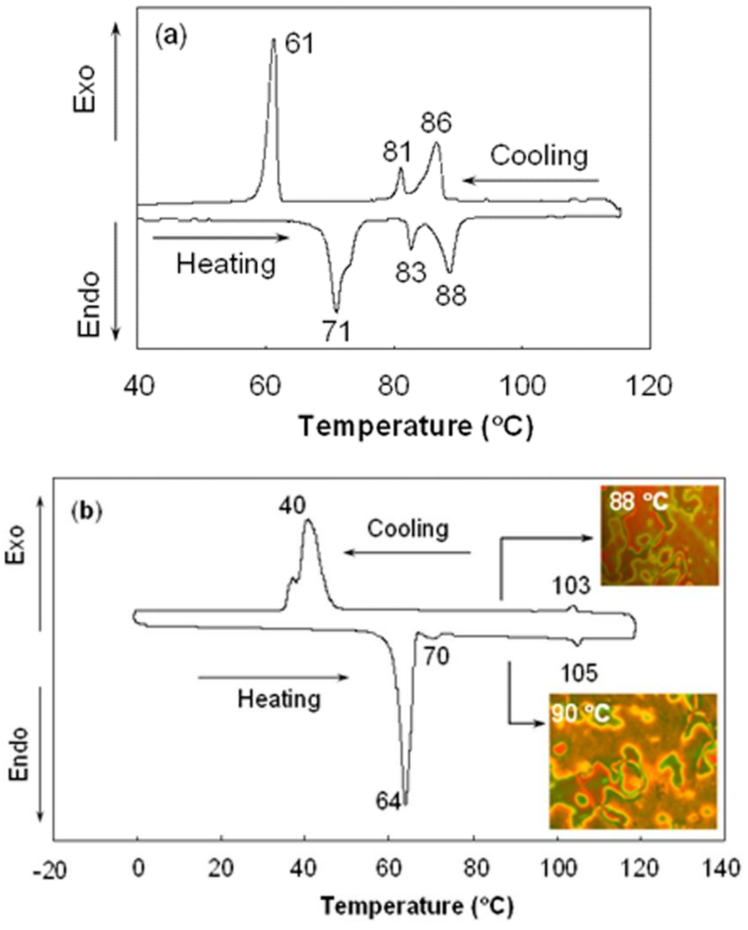

Supporting Figure 3 Differential scanning calorimetry curves and polarizing optical microscope images of (a) DA11AB and (b) the mixture of DA11AB and C9A in the molar ratio of 1:4 on the heating and the cooling (5 ℃ min^{-1}). Pure DA11AB only shows a very narrow nematic phase window of 5 ℃. After blended with C9A in the optimized proportion, the nematic phase window was greatly enlarged to 63 ℃.

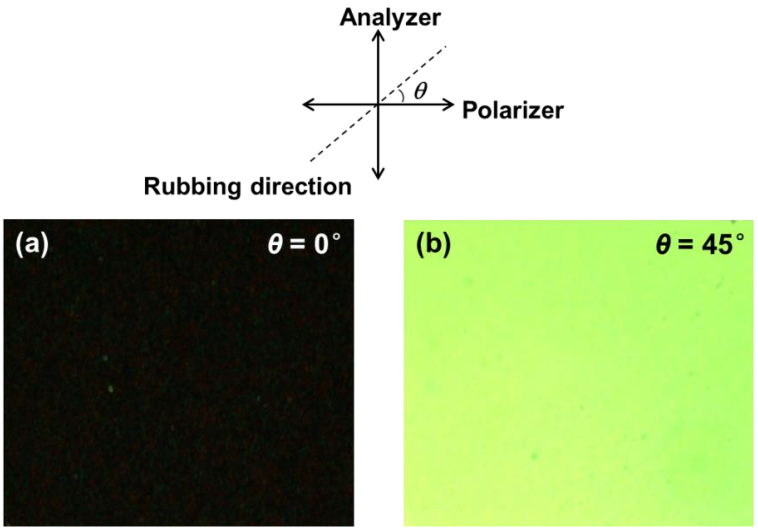

Supporting Figure 4 Polarizing optical microscope images of the LDLCF. θ denotes the angle between the rubbing direction of the films and the polarization direction of either polarizer.

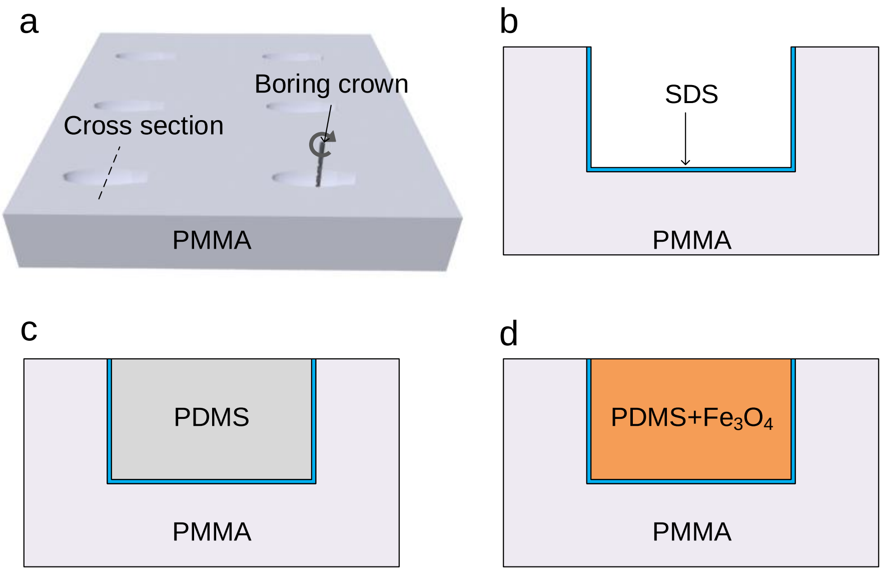

Supporting Figure 5 Processes to make the head of the swimming robot. (a) Engraving mold on a PMMA board by engraving machine. (b) Cross section of the mold and SDS coated on the surface of the mold. (c) Injecting PDMS pre-polymer in the mold to make upper layer of the head. (d) Injecting PDMS pre-polymer and Fe_3O_4 in the mold to make bottom layer of the head.

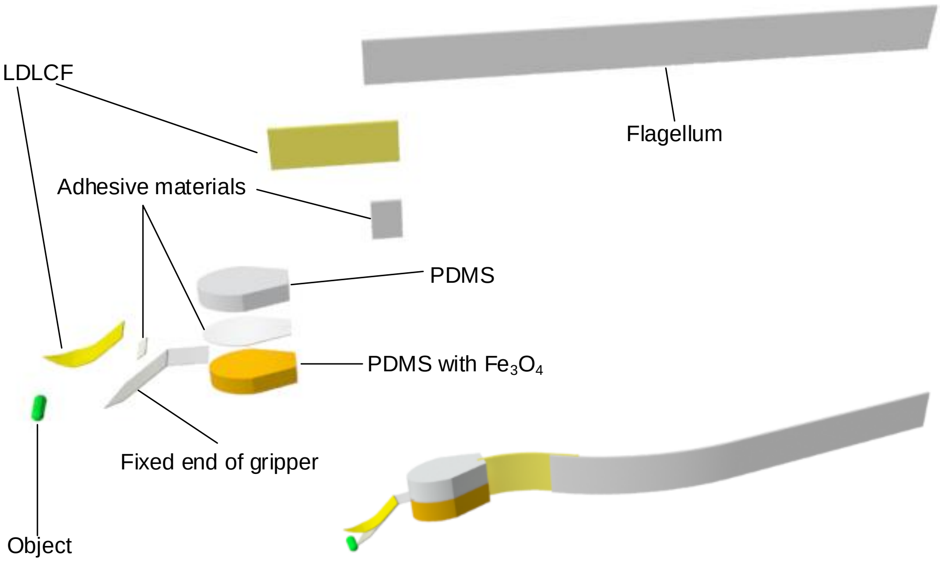

Supporting Figure 6 Parts of the swimming robot. The bottom-right insert is the assembled swimming robot.

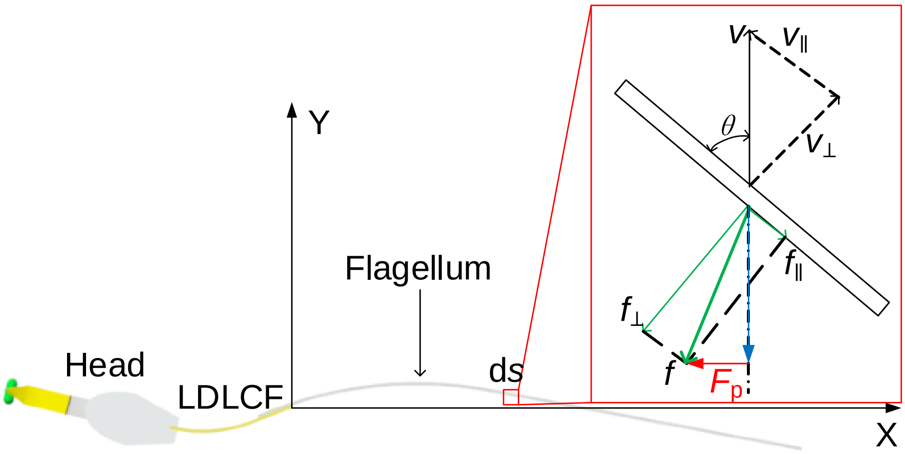

Supporting Figure 7 Generation mechanism of propulsive force. As the viscous coefficients parallel and perpendicular to the flagellum are different, the resultant viscous force has a component force which is propulsive force.

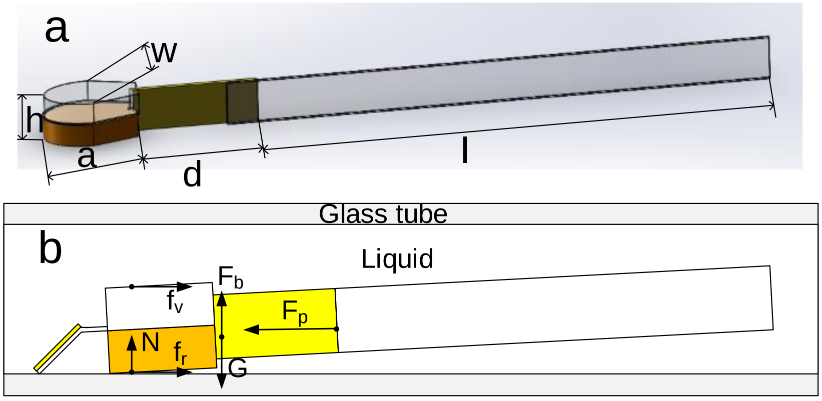

Supporting Figure 8 Dimension and force analysis of the swimming robot. (a) Dimension of the swimming robot (b) Force analysis in the liquid.

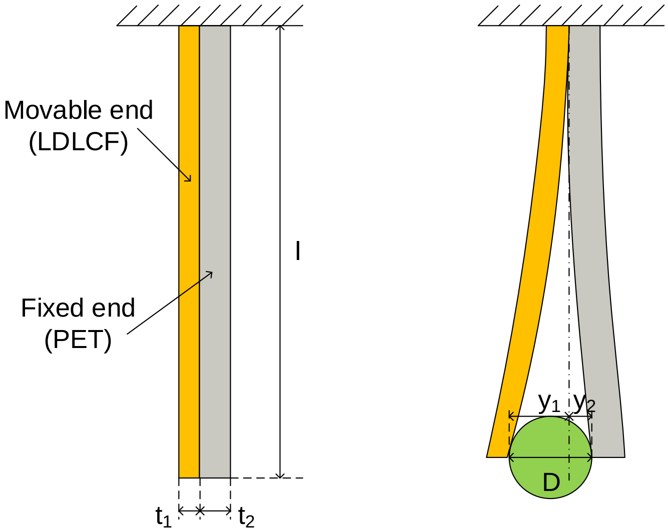

Supporting Figure 9 Schematic diagram of closed gripper and grabbed object. The gripper is made up of movable end and fixed end. The sum of bending deflection equals to the diameter or thickness of the grabbed object.
